# Salvage Laparoscopic-Assisted Anorectoplasty after Failed Vestibular Fistula Repair Using Magnetic Resonance Image Guidance

**DOI:** 10.1055/s-0039-1688486

**Published:** 2019-05-23

**Authors:** Matthew W. Ralls, Brian P. Fallon, Maria Ladino-Torres, Peter F. Ehrlich, Marcus D. Jarboe

**Affiliations:** 1 Section of Pediatric Surgery, Department of Surgery, C.S. Mott Children's Hospital, University of Michigan, Ann Arbor, Michigan, United States; 2 Section of Pediatric Radiology, Department of Radiology, University of Michigan Medical School and C.S. Mott Children's Hospital, Ann Arbor, Michigan, United States; 3 Division of Interventional Radiology, Department of Radiology, C.S. Mott Children's Hospital, University of Michigan, Ann Arbor, Michigan, United States

**Keywords:** posterior sagittal anorectoplasty, magnetic resonance imaging, image guidance, anorectal malformation, rectovestibular fistula

## Abstract

Patients with vestibular fistula have a good functional outcome after posterior sagittal anorectoplasty (PSARP). While continence is often preserved, close follow-up and management of constipation are often required. Redo anorectal surgery has been associated with worse functional outcomes compared with primary procedures, possibly due to injury and scarring of the pelvic floor musculature and sphincter complex. Our group has a growing experience in the use of intraoperative real-time magnetic resonance imaging (MRI) for anorectal malformation repairs. We present a case of salvage operation of a failed PSARP for vestibular fistula.

## Introduction

Anorectal malformation (ARM) occurs in 1.4 to 5.7 per 10,000 live births, depending on which region of the world is studied, 1 of which vestibular fistulae compose approximately 10%. 2 Posterior sagittal anorectoplasty (PSARP) is the operation of choice and the standard of care. These children have potential for excellent bowel control, 3 though constipation is common postrepair, and over half of the children will need continued bowel management. 4 5 While little data are available for functional outcomes after revision surgery for ARM patients, redo pelvic surgery in the pediatric population for other indications is associated with worse outcomes. 6 7 8 Our group has a growing experience in the use of intraoperative real-time magnetic resonance imaging (MRI) for ARM repairs. 9 10 11 Here, we describe the use of MRI to guide a repair after failed PSARP for vestibular fistula in an attempt to reduce dissection injury to an already compromised pelvis.

## Clinical Presentation

The patient is a 20-month-old female with a past medical history of DiGeorge's syndrome with associated interrupted aortic arch, ventricular septal defect, and rectovestibular fistula. The child had a normal sacral ratio and an otherwise negative VACTERL (vertebral defects, anal atresia, cardiac defects, tracheoesophageal fistula, renal anomalies, and limb abnormalities) work-up. After repair and recovery from cardiac surgery, the patient was taken for PSARP at 1 year of life. The initial recovery period was uneventful, and she was sent home on the third postoperative day. On postoperative day 8, a dehiscence of the anoplasty was noted on examination and a diverting loop sigmoid colostomy was subsequently performed. She was then referred to our colorectal clinic for management.

**Fig. 1:**
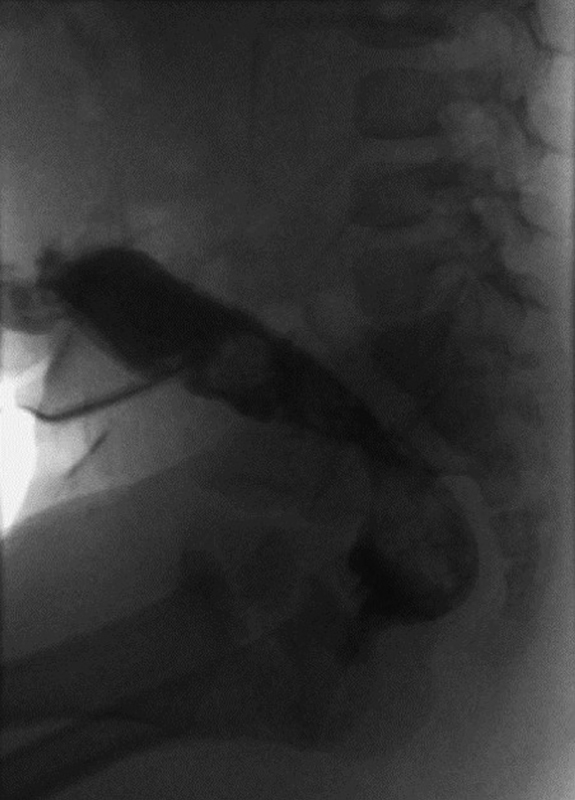
Distal colostogram following failed posterior sagittal anorectoplasty for rectovestibular fistula.

**Fig. 2:**
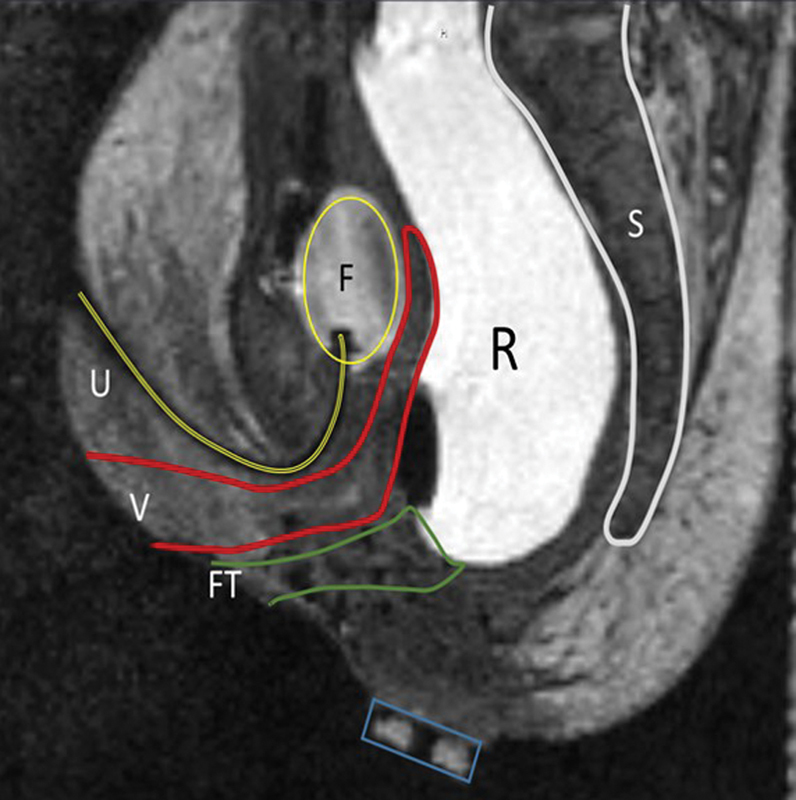
Sagittal magnetic resonance image (MRI) of the patient with failed primary posterior sagittal anorectoplasty for vestibular fistula. F, Foley; FT, previous fistula tract; R, rectum; S, sacrum; U, urethra; V, vagina.

The patient was allowed a period of several months to recover from the initial operation and diverting colostomy. Nutritional support was necessary to treat failure to thrive. Once healthy, a distal colostogram was performed to better understand the anatomy of the mucus fistula and retracted anoplasty. This was done in a manner similar to that of a standard work-up for a high male ARM ( Fig. 1 ). While no marker was placed at the site of the neoanus, the colostogram resembled a high lesion in a male with ARM. The patient was taken to the intraoperative MROR suite (IMRIS) equipped with a 1.5T Magnetom Espree magnet (Siemens, Erlangen, Germany). She was positioned in the supine position with legs suspended using the previously described suspension system. 10 A muscle stimulator was used to identify and mark the superficial external sphincters. An initial MRI scan was performed to delineate pelvic anatomy, which was reviewed with a radiologist on site in the operating room (OR). The imaging was remarkable for postoperative changes along the former incision from the superficial subcutaneous tissue to the level of the pelvic floor muscles ( Fig. 2 ). While in the same position in the OR, an MRI-compatible needle (MR Bard Ghiatas Beaded Breast Localization Wire, 20 g × 7 cm, Bard, Tempe, Arizona, United States) was passed through the sphincter complex from skin to above the levators with real-time MRI guidance. 9 A pediatric surgeon trained in interventional radiology (author M. D. J.) performed the needle placement, and the diagnostic pediatric radiologist provided assistance with image acquisition, sequence selection, and assessment of anatomy and needle location. The retracted neoanus was deep in the pelvis, as shown in Fig. 2 ; however, the colon was easily mobilized laparoscopically and readied for redo pull-through. The needle was used to guide dilation of a tract through which the neorectum was pulled to create a new anastomosis, as described previously. 9

The child recovered well from the operation and was discharged on the fourth postoperative day. A colostomy takedown and Heineke–Mikulicz-like stricturoplasty 12 was performed 4 months after the redo pull-through, which was uneventful. At the latest follow-up, 1 year after her redo pull-through, the patient was experiencing moderate constipation. On a bowel regimen of senna and fiber, she was having three to five soft bowel movements per day.

## Discussion

Comprehensive care of patients with ARM has been advanced over the last several decades, yet functional outcomes can still be improved. The application of MRI in the diagnosis, understanding, and treatment of ARM may provide major advancements toward allowing these children to live more normally through toilet training and beyond. MRI not only looks at the current standard of care in predictive measurements (e.g., quality of the sacrum, spinal cord lesions, and level of the fistula) but can also evaluate the pelvic musculature directly. Furthermore, use of MRI during the operative repair allows for the anatomically correct positioning of as many muscle groups and fibers as possible. We believe that this will benefit the children's long-term continence, though outcome data are still forthcoming. In this case, our institutional experience in treating primary male high ARM lesions with real-time MRI allowed us to perform a minimally invasive redo procedure in a female with a rectovestibular fistula. This operation did not require a new perineal incision—only the laparoscopy incisions. This made for an easy recovery and less chance for the wound complications often seen with redo surgery.

**Fig. 3:**
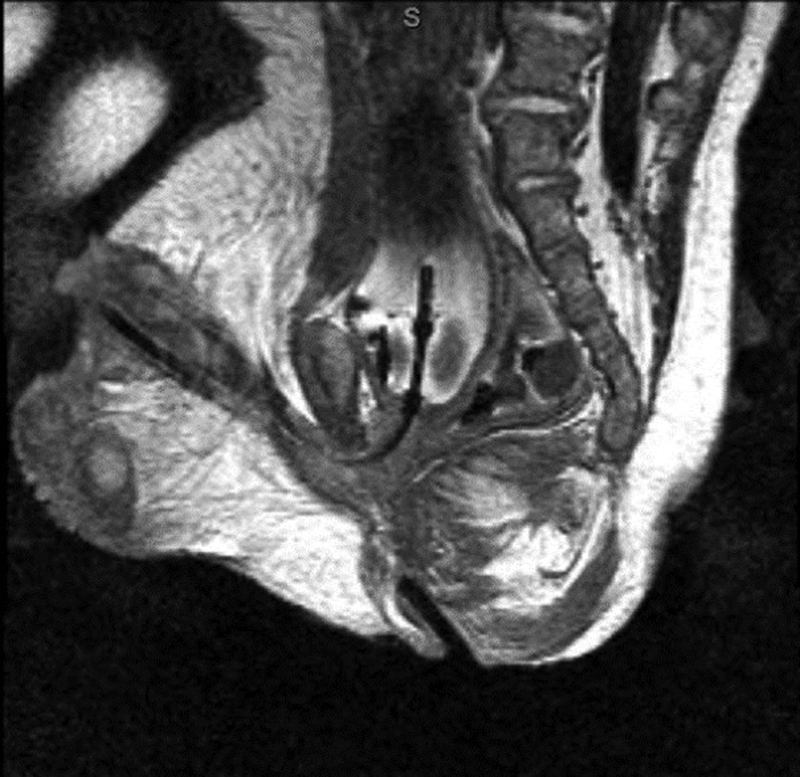
Sagittal magnetic resonance image (MRI) showing the superficial sphincter and deep sphincter complex, as well as the levator muscles. Note a Foley catheter balloon within the bladder as well as an MR-compatible needle within the first 1.5 cm of the sphincter complex.

A sagittal view of the patient shown in Fig. 2 is remarkable for the difficulty in delineating sphincter complex from postoperative scarring. This highlights the tissue destruction and distortion noted after a standard PSARP, which severely inhibits our interpretation of pelvic floor musculature. The sagittal view alone does not define the sphincter complex well. However, a full three-dimensional (3D) T2 scan is completed before needle placement. This preliminary sequence is used to pick the position of the ideal real-time sagittal plane. The sagittal view from the real-time sequence, and the sagittal, axial, and coronal views from the initial T2 sequence are used to identify the path of the sphincters. The sagittal view is the most effective in guiding the needle in real time after the path is described with the static images. For comparison, a preoperative MRI of the pelvis from a different patient is shown in Fig. 3 . This is a male with bulbar fistula without associated anomalies. The image serves to show the details in the pelvic structures and specifically the musculature. Here the superficial sphincter complex is easily appreciated, but the deep sphincters and levators can also be seen. An experienced pediatric radiologist with expertise in MRI and a urogynecologist who specializes in MRI of the sphincter complex in adults are present for the MRI portion of the procedure to assist with image interpretation. Despite the relative lack of precision in MRI detail in this patient, the needle was placed through the center of the complex as confirmed through axial imaging. The scarring from the dissection of the previous operation clearly has implications on the sphincter muscle and possibly on long-term sphincter function. However, using both the preliminary 3D sequences and the real-time sequence, we can discern the anatomically correct path for the neorectum. This technique saved a second open dissection and the resultant cautery injury to the external sphincter complex as well as the pelvic floor. We hypothesize that this will result in better long-term function. This will be difficult to prove immediately based on the individual nature of each of the malformations and the time needed to follow long-term outcomes.

## Conclusion

Performing redo anorectoplasty under image guidance can mitigate the difficult dissection required in redo pelvic operations. MRI safely delineates the pelvic floor and sphincter musculature. Needle placement and dilation avoid the repeated pelvic trauma from electrocautery, thus decreasing the resultant injuries to pelvic muscles and nerves. The patient is also spared a second perineal incision and the concomitant risks of wound complications. Image-guided techniques provide a less invasive approach and may preserve more muscle and nerve tissue, leading to improved long-term functional outcomes for ARM patients needing reoperative pelvic surgery.

## Notes

New Insights and the Importance for the Pediatric Surgeon Image-guided approaches to ARM repair provide a less invasive approach and may preserve more muscle and nervous tissue, leading to improved long-term functional outcomes for ARM patients needing reoperative pelvic surgery.
